# Improving diagnostics and surveillance of malaria among displaced people in Africa

**DOI:** 10.1186/s12939-025-02378-6

**Published:** 2025-01-21

**Authors:** Toufik Abdul-Rahman, Oyinbolaji Akinwande Ajetunmobi, Gafar Babatunde Bamigbade, Innocent Ayesiga, Muhammad Hamza Shah, Tolulope Sharon Rumide, Abdurahman Babatunde Adesina, Ganiyat Adekemi Adeshina, Oluwabusola Elizabeth Oni, Bet-ini Nsikak Christian, Abdullahi Tunde Aborode, Andrew Awuah Wireko, Hala Ibrahim Thaalibi, Iman Mustafa Abdalla, Sewar Basheer Banimusa, Justice Ndubuisi Jonathan, Isreal Ayobami Onifade, Md Ariful Haque

**Affiliations:** 1 Department of Research, Toufik’s World Organization, Anonova 10, Sumy, 40007 Ukraine; 2 Master of Public and International Affairs, University of Lagos, Akoka, Nigeria; 3 Department of Food Science, College of Agriculture and Veterinary Medicine, United Arab Emirates University, Al-Ain, Abu Dhabi, United Arab Emirates; 4 Research Department, Ubora Foundation Africa-Uganda, Kampala, Uganda; 5 School of Medicine, Queen’s University Belfast, Belfast, UK; 6 Department of Microbiology, University of Ilorin, Ilorin, Nigeria; 7 Department of Veterinary Public Health, Faculty of Veterinary Medicine, Usmanu Danfodiyo University, Sokoto, Nigeria; 8 Department of Pharmacology, Therapeutics and Toxicology, Faculty of Basic Medical Sciences, University of Lagos, Lagos, Nigeria; 9 Hospitals Management Board, Uyo, Akwa Ibom State Nigeria; 10 Healthy Africans Platform, Research and Development, Ibadan, Nigeria; 11 Faculty of Medicine, Beirut Arab University, Beirut, Lebanon; 12 Faculty of Medicine and Health Science, Bakhtalruda University, Dueim, Sudan; 13 Basic Medical Sciences, Yarmouk University, Irbid, Jordan; 14 Faculty of Pharmaceutical Sciences, Abia State University, Isuikwuato, Nigeria; 15 Department of Biological Sciences, University of Albany, SUNY, Albany, USA; 16 Department of Public Health, Atish Dipankar University of Science and Technology, Dhaka, Bangladesh

**Keywords:** Malaria, Displaced people, Africa, Diagnostic, Surveillance

## Abstract

African communities that have been forced to leave their homes experience a considerably greater susceptibility to malaria as a result of densely populated living conditions, restricted availability of healthcare, and environmental influences. Internally displaced individuals frequently live in large settlements with restricted availability to drinking water, essential sanitation, and medical services, intensifying the spread of malaria. As a result, the occurrence of malaria is significantly more common among refugees and internally displaced individuals compared to those who are not displaced. This leads to greater rates of illness and death, especially among young people. Insufficient monitoring worsens the condition, leading to delayed identification and medical intervention, and contributing to a higher incidence of severe malaria and deaths. Furthermore, these communities are faced with economic consequences that contribute to the continuation of poverty and the worsening of socio-economic inequalities. Furthermore, the psychological impact of malaria, which is marked by feelings of anxiety and uncertainty, is particularly severe in vulnerable populations such as displaced children and pregnant women, aggravating the overall burden. Hence, addressing malaria in displaced populations in Africa requires comprehensive and well-coordinated strategies. Advanced diagnostic and surveillance technologies are essential for promptly identifying and treating malaria, providing chances to monitor and control its spread effectively. Collaboration among healthcare, policy, and humanitarian sectors is crucial for implementing comprehensive solutions that incorporate enhanced diagnostics, surveillance, and socio-psychological support. Active involvement of the community, usage of Community Health Workers, and regular collection of surveillance data are crucial in increasing awareness, directing control efforts, and tackling the specific difficulties encountered by displaced groups. Moreover, the implementation of environmental management, the incorporation of health services, and the utilization of adaptable healthcare interventions are essential for reducing the effects of malaria. To mitigate the impact of malaria and improve health outcomes among displaced populations in Africa, it is crucial to focus on these specific areas.

## Introduction

Malaria is a deadly tropical disease spread by a parasitic vector called Plasmodium. Over the years, malaria continues to rank among the top infectious diseases implicated globally in causing high morbidity and mortality in low- and middle-income countries, especially in Africa [1]. Recently, most non-endemic countries have been challenged with a significant increase in imported malaria due to the influx of travelers, immigrants, and displaced refugees from predominantly endemic countries [2, 3]. Malaria ranked as the second foremost cause of infectious disease-related mortality after human immunodeficiency virus/acquired immune syndrome (HIV/AIDS) [4]. It has also consistently topped the list of major diseases responsible for high morbidity and mortality in most of Africa, especially sub-Saharan Africa [5]. Statistically, an estimated 1.24 million malaria-related deaths, which are mostly children, occur annually in sub-Saharan Africa [6]. Taxonomically, the World Health Organization (WHO) has named five Plasmodium species (*P. falciparum, P. ovale, P. malariae, P. knowlesi, and P. vivax*) as the vectors implicated in human malaria with P. falciparum being the most virulent and common species in Africa. Epidemiologically, malaria exerts significant challenges on pregnant women and children causing malaria-induced anemia, fetal low birth weight, and impairing the normal cognitive and social development in children [7]. Also, malaria accounts for the estimated daily death toll of approximately 3000 children (5 years and below), making it the first leading cause of child mortality [4]. Severe malaria has also been implicated in causing mental and physical health retardation among children survivors [7]. To minimize this uprising malaria-induced morbidity and mortality, especially the one that originated from *P. falciparum*, researchers have identified timely, precise, and accurate diagnosis as well as prompt treatment to be the gold standard [8].

Undoubtedly, a range of extensive measures implemented to combat this harmful challenge that has been affecting developing nations for many years have achieved significant outcomes in reducing malaria in Africa. However, recent instances of violent conflicts in these countries have led to a scarcity of medical resources, socio-economic destruction, and a rapid rise in the number of displaced individuals, worsening the problem of overpopulation [9]. These factors ultimately heighten the danger and opportunity for the transmission, diagnosis, and extensive monitoring of malaria among individuals living in refugee camps. Researchers have found a connection between the high occurrence and susceptibility to malaria diseases and various factors such as socioeconomic disruptions, inadequate healthcare systems, and infrastructure. These factors collectively contribute to malaria being the primary cause of death in countries where it is prevalent [10].

In recent years, there has been a significant increase in the occurrence of malaria among Internally Displaced Persons (IDPs) in Africa. This poses a new challenge for humanitarian efforts aimed at assisting displaced individuals [9]. Despite malaria being a prominent infectious disease among displaced communities in Africa, epidemiological data and statistics on its prevalence are scarce. The main goal of any epidemiological study is to reduce and prevent unneeded deaths and illnesses. Therefore, it is necessary to examine the current epidemiology of malaria among IDPs in Africa in order to enhance its diagnosis and monitoring. This can contribute to the development of a more efficient and effective control system, as well as the provision of essential healthcare services [9]. This review aims to assess the existing surveillance and diagnostic methods, difficulties, and consequences of malaria in displaced populations in Africa.

## Methodology

This review assesses the application of diagnostic and surveillance tools for malaria among displaced people in Africa from 2000 to 2023. Full-text articles written in English were included to ensure a comprehensive evaluation of both established practices and significant advancements over this period. An exhaustive literature search was conducted using PubMed and Scopus. Key search terms included “malaria diagnostics,” “malaria surveillance,” “displaced populations,” “refugees,” “malaria transmission,” and “malaria control” (Table 1). Additional sources were identified through a manual search of references cited in recent disease-specific reviews and authoritative bodies such as the WHO.

**Table 1:** Summary table for the methodology of this review

Methodology Steps	Description
Literature Search	PubMed and Scopus
Inclusion Criteria	● Articles written entirely in English ● Emphasis on practical uses in the fields of malaria diagnostics and surveillance
Exclusion Criteria	● Studies that have not been published ● Presentations in poster format ● Conference or stand-alone abstracts ● Case reports
Search Terms	Keywords such as “malaria diagnostics,” “malaria surveillance,” “displaced populations,” and “refugees” coupled with indicators like "malaria transmission" and "malaria control."
Additional Search	● Thorough inspection of references mentioned in recent reviews focused on specific diseases through manual examination ● Websites of authoritative bodies such as the WHO ● No predefined restriction on the number of studies to be considered ● Inclusive of a variety of study designs: *● Descriptive studies* *● Field studies* *● Cohort studies* *● Observational studies*

**Fig. 1:**
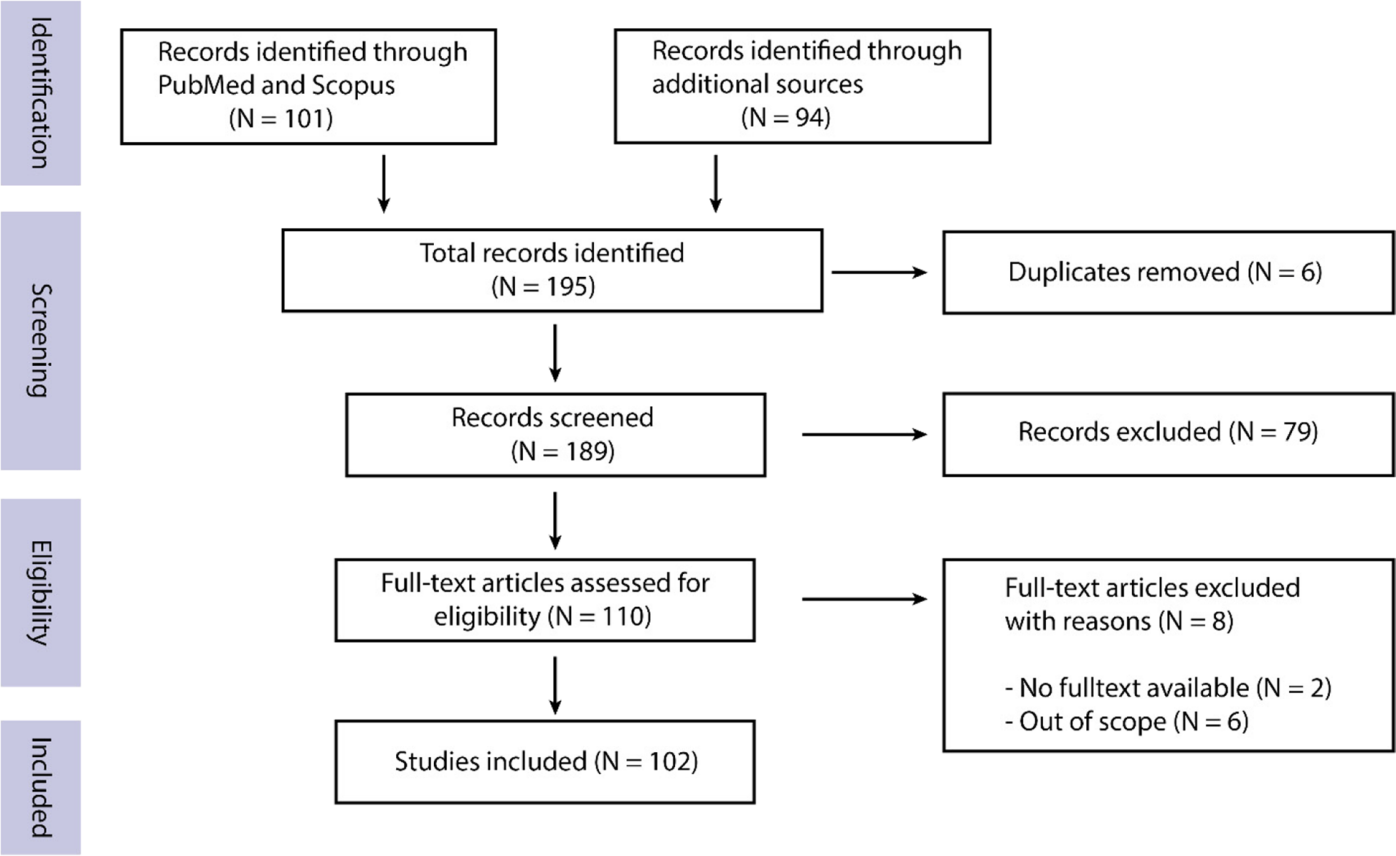
PRISMA Flow-diagram

The Preferred Reporting Items for Systematic Reviews and Meta-Analyses (PRISMA) guidelines was used to identify and include studies based on eligibility criteria (Fig. 1) [11]. Exclusion criteria were set to maintain high-quality evidence, excluding non-peer-reviewed studies, poster presentations, conference abstracts, and case reports. No limit was set on the number of studies included, allowing for a thorough review. Various study designs were considered, including descriptive, field, cohort, and observational studies, providing a broad perspective on malaria diagnostics and surveillance among displaced populations in Africa. Two authors reviewed and meticulously screened the retrieved articles, and addressed discrepancies with the help of a third author.

## Malaria among displaced people in Africa

### Background and overview of displacement in Africa

Displaced populations are one of the most vulnerable population categories as they face limitations, such as access to proper healthcare, sanitation services, ideal food, and clean water [12]. They live in an overcrowded manner and hence are more predisposed to surges in malnutrition, malaria, and HIV infection [13]. The 2020 report by the United Nations High Commissioner for Refugees (UNHCR) shows that the number of refugees and IDPs amounted to an unforeseen 82.4 million globally, of which 31.8 million (38%) were seen in Africa [14]. Children in Sub-Saharan African countries below 18 years of age accounted for 57% of this concerned population, with rates higher as 62–63% in South Sudan, the Central African Republic, the Democratic Republic of Congo (DRC), and Somalia [14]. Displacement in African countries has been due to factors like political violence, terrorism, banditry, environmental calamities like floods, and escalated events like drought and famine emergencies [15].

### Prevalence of malaria in displaced populations

Malaria continues to be a problematic condition in the tropics, especially in complex humanitarian emergencies (CHEs) and conflict sites accommodating internally displaced people (Table 2) [4, 8, 9, 12, 16–22], (Table 3) [4, 8, 9, 12, 16–23]. It's one of the main causes of mortality in many developing countries today [1]. Reproductive women and children make up the majority of displaced populations and are both at the highest risk of severe malaria and mortality [24]. Besides, malaria stands as the second leading source of mortality among infectious diseases in Africa, second only to human immunodeficiency virus/acquired immune syndrome (HIV/AIDS) [25]. The Plasmodium falciparum type of malaria is a major health challenge for displaced populations, especially in sub-Saharan Africa [26], accounting for 26% to 31% of mortality in immuno-compromised refugee children annually [27]. This higher prevalence of malaria in African displaced populations presents an emerging challenge in humanitarian response [9].

**Table 2:** Overview of case studies on malaria among displaced populations in Africa

Case Study Location	Population	Intervention	Key Outcomes	Authors
Sofala and Cabo Delgado, Mozambique	Displaced persons post-cyclone and violence	Surveillance activities, RDT administration	Identified a 4.5% malaria-positive rate among screened individuals; highlighted the need for combined diagnostic algorithms due to COVID-19 and malaria symptom overlap	Di Gennaro et al. 2022
Northern Zambia	Congolese refugee children	Surveillance data and hospital record analysis	Similar malaria prevalence in refugee settlements and high-burden areas; refugee children from transit centers had better outcomes than those from permanent settlements	Hauser et al. 2022
Eastern DRC, IDP camp	100 Febrile Children Under 5 years of age from the IDP camp	Active case detection of household contacts	Active case-finding was not efficient; symptom-based screening was suggested for identifying at-risk individuals	Hamze et al. 2016
Displacement camp, DRC	Children living in displacement camp	Cross-sectional surveys, RDTs	Higher prevalence of P. falciparum infection in camp children compared to nearby village; highlighted low bed net usage	Charchuk et al. 2016
General Hospitals in Ebonyi State, Nigeria	Patients with malaria symptoms	Comparison of diagnostics	Showed variability in diagnostic accuracy; recommended combined use of microscopy and RDT	Ugah et al. 2017
NorthWest, Central African Republic (CAR)	Community through CHWs	Malaria RDTs and ACT treatment	High positivity rate; effective treatment with ACT for positive cases	Ruckstuhl et al. 2017
Ardamata IDP camp, Sudan	Patients with suspected malaria	Nested PCR for malaria detection	High malaria prevalence; highlighted gender as a risk factor	Eshag et al. 2020
Non-endemic countries	Suspected malaria in travellers, migrants, refugees	Novel FRET-qPCR diagnostic	Achieved high accuracy in species identification; suggested for routine clinical use	Schneider et al. 2021
South Sudan	Refugees, returnees, internally displaced people	Implementation of WHO-recommended malaria control interventions per the National Malaria Strategic Plan	Despite challenges, and progress in implementing malaria control interventions; the need for enhanced coordination, infrastructure, and resource allocation highlighted	Pasquale et al., 2013
North Wollo zone, Ethiopia	Patients, pregnant women, elders, community and religious leaders, health professionals	Use of traditional medicines, medications from unaffected areas, home-to-home healthcare	The conflict led to a breakdown in the health system, affecting the provision and utilization of health services, including those for malaria. Coping strategies were developed in response	Arage et al., 2023
Eight countries in sub-Saharan Africa Cameroon, DRC, Ghana, Nigeria, Senegal, Sierra Leone, Tanzania, and Uganda)	Community health workers, health center managers, parents of children receiving chemoprevention, national decision-makers	SMC and IPTi	SMC and IPTi are largely accepted but face challenges like children's absenteeism, parents' reluctance, access to water, and staff turnover. Identified drivers and barriers to acceptance	Audibert & Tchouatieu, 2021

*Abbreviations: DRC* Democratic Republic of Congo, *SMC* Seasonal Malaria Chemoprevention, *RDT* Rapid Diagnostic Tests, *PCR* Polymerase Chain Reaction, *IDP* Internally Displaced Persons

**Table 3:** Methodology overview of studies on malaria among displaced populations

Study Location	Study Design	Participant Selection	Diagnostic Methods	Key Metrics Assessed	Authors
Sofala and Cabo Delgado, Mozambique	Surveillance study	IDP sites in Sofala and Cabo Delgado	RDTs, assessment of COVID-19 symptoms, HIV risk	Malaria prevalence, COVID-19 symptom prevalence, HIV screening referral rates	Di Gennaro et al. 2022
Northern Zambia	Malaria prevalence, COVID-19 symptom prevalence, HIV screening referral rates	Refugee and local children with severe malaria	Multivariable regression models, geospatial visualization	Malaria prevalence, in-hospital mortality, malnutrition rates	Hauser et al. 2022
Eastern DRC, IDP camp	Prospective community-based survey	100 Febrile Children Under 5 years of age from the IDP camp	Malaria case detection efficiency, symptom-based screening effectiveness	Malaria case detection efficiency, symptom-based screening effectiveness	Hamze et al. 2016
Displacement camp, DRC	Cross-sectional surveys	Children in camp vs. nearby village	RDTs for P. falciparum, demographic and clinical data collection	P. falciparum prevalence, febrile illness attributable to malaria	Charchuk et al. 2016
General Hospitals in Ebonyi State, Nigeria	Comparative study	Patients with clinical malaria symptoms	Microscopy, RDT, nested PCR	Diagnostic accuracy of microscopy vs. RDT	Ugah et al. 2017
NorthWest, Central African Republic	Retrospective review of patient registers	Consultations with CHWs	RDT	Malaria positivity rates, treatment with ACT	Ruckstuhl et al. 2017
Ardamata IDP camp, Sudan	Cross-sectional study	Patients with suspected malaria	Nested PCR	Malaria prevalence, species identification	Eshag et al. 2020
Non-endemic countries	Evaluation of diagnostic tool	Travelers, migrants, refugees with suspected malaria	FRET-qPCR	Diagnostic accuracy, species identification	Schneider et al. 2021
South Sudan	In-depth appraisal according to WHO standard procedures	Documents on malaria control, internal assessments, and field reviews by external teams	Not specified	Progress and challenges in malaria control interventions, resource constraints, health system evaluation	Pasquale et al., 2013
North Wollo zone, Ethiopia	Descriptive qualitative study	100 purposively selected participants (patients, pregnant women, elders, community/religious leaders, health professionals)	Not specified (qualitative interviews and focus groups)	Health consequences of conflict, including the impact on health services and infrastructure, coping strategies for healthcare	Arage et al., 2023
Eight countries in sub-Saharan Africa Cameroon,DRC, Ghana, Nigeria, Senegal, Sierra Leone, Tanzania, and Uganda)	Qualitative, in-depth interviews	179 participants including CHWs, health center managers, parents, and national decision-makers	Thematic analysis of transcribed verbal responses	Perceptions and attitudes towards SMC and IPTi, challenges to coverage and acceptance, efficacy perceptions	Audibert & Tchouatieu, 2021

*Abbreviations: DRC* Democratic Republic of Congo, *SMC* Seasonal Malaria Chemoprevention, *RDT* Rapid Diagnostic Tests, *PCR* Polymerase Chain Reaction, *IDP* Internally Displaced Persons, *CHW* Community Health Workers

#### Comparison with non-displaced populations

According to a study on Malaria epidemiology in Congolese refugee children living in holoendemic areas of northern Zambia, it was established that malaria tests were more positive in refugees than non-refugees brought to the same hospital [24]. This is attributed to factors, such as differences in nutrition or immune status, housing situations, healthcare, and nearness to mosquito-breathing areas. Other challenges such as delayed care following exposure to mosquito bites were identified and these aggravated the exposure, and higher parasitemia in refugee children compared to local children who may have similar spatial predisposition [24]. Furthermore, following a community-based survey of averagely symptomatic children and febrile children brought to the health clinic, the point prevalence of Plasmodium falciparum was substantially greater among children living in an IDP camp compared to neighboring village controls [28]. Moreover, studies have also confirmed that the psychological and physical health of displaced populations is significantly poorer than non-displaced populations [28]. Most of these factors demonstrate the increased chances of malaria predisposition among displaced populations necessitating continuous efforts and strategies.

### Factors contributing to malaria transmission in displaced populations

Displaced populations such as refugees and IDPs, represent a critical challenge in public health. Brooks et al. and Hamissou et al. emphasize the heightened risk these populations face due to their living conditions and circumstances. The temporary settlements they inhabit often lack essential infrastructure, proper sanitation, and effective vector control measures, creating favorable conditions for the survival of mosquitoes and malaria transmission [10, 29, 30]. Also access to vital preventive interventions, including insecticide-treated bed nets and indoor residual spraying (IRS), is frequently hindered in such settings [31]. Furthermore, conducting robust malaria surveillance and data collection within these highly mobile and resource-constrained communities is challenging [32, 33]. Connolly et al. and The Sphere Project highlight a myriad of interconnected risk factors, for example, mass population movements, overcrowding, economic and environmental degradation, and poverty as significant in malaria transmission. Other factors demonstrated include inadequate access to safe water, poor sanitation, limited healthcare facilities, and inadequate waste management [34, 35]. Thus, addressing these multifaceted factors necessitates a comprehensive and coordinated approach that acknowledges the unique vulnerabilities of displaced populations and considers the interplay of environmental, socioeconomic, and healthcare-related determinants.

#### Environmental and living conditions

The displacement camps provide the ideal conditions for the spread of malaria, as they create an atmosphere that is conducive to the breeding and multiplication of malaria-carrying insects. Populations displaced by conflict or natural disasters often experience higher rates of infectious diseases, such as malaria. This can be attributed to factors such as poor living conditions, the movement of people without defense to areas where the disease is common, and limited access to healthcare [24, 36]. The displacement settings are commonly marked by densely populated living conditions, where temporary shelters fail to provide sufficient protection against Anopheles mosquitoes. Additionally, there are inadequate drainage systems that lead to the accumulation of stagnant water, which creates ideal breeding grounds for these disease-carrying insects [10, 37]. The proximity of displacement camps to areas with high malaria transmission rates increases the likelihood of infection among these susceptible groups [38].

The environmental constraints encompass problems concerning cleanliness, restricted availability of uncontaminated water, and the absence of extensive use of vector control measures like insecticide-treated nets (ITNs) and IRS [39, 40]. Instances from both the past and present in different areas highlight the increased susceptibility of displaced populations to the negative effects of malaria, such as illness and death. This vulnerability has been observed among Afghan refugees, Kurdish refugees in Iraq, IDPs in Uganda, and displacement camps in the Democratic Republic of the Congo (DRC) [24, 33, 41, 42]. The DRC has one of the highest malaria loads in the world while facing substantial socio-economic issues and shortcomings in its healthcare system. Even though attempts have been made to provide bed nets, children in displacement camps still have notably high rates of malaria [33, 43, 44]. This emphasizes the necessity of a comprehensive approach to controlling malaria in areas where people have been displaced, which includes combining medical, environmental, socio-economic, and infrastructural measures. Understanding the complex relationship between environmental circumstances, vector populations, and human variables is crucial for developing successful and long-lasting strategies to reduce malaria transmission in high-risk populations [45].

#### Access to prevention and treatment

This factor is critical in mitigating malaria transmission among displaced populations in Africa, yet significant barriers persist. The WHO advocates for the use of ITNs, intermittent preventive treatment (IPT), and effective management of clinical malaria as key interventions [46]. However, the deployment and use of ITNs are often less than ideal in displacement contexts, leaving many at risk [47]. Disruptions in healthcare services, notably in antenatal care, hinder the provision of IPT for pregnant women in refugee settings, while the execution of seasonal malaria chemoprevention (SMC) faces obstacles in regions with high displacement [48, 49].

The challenges extend to diagnostic and treatment services, where access to rapid diagnostic tests (RDTs) and artemisinin-based combination therapies is compromised by health system limitations, supply shortages, and interruptions in healthcare provision [50, 51]. Despite the acknowledged efficacy of ITNs and IPT, issues such as low ITN utilization even when distributed for free, inadequate coverage of national prevention strategies, the critical role of individual awareness and health system effectiveness for adoption, and the need for better antenatal and community health approaches are evident across countries like Kenya, Cameroon, Tanzania, and Uganda [52–55]. This necessity for targeted efforts to enhance access to and use of malaria prevention and treatment services among displaced populations, addressing both supply-side constraints and demand-side factors to curb malaria transmission effectively.

#### Surveillance and data collection challenges

Efficient monitoring and detailed data gathering are crucial for comprehending and tackling the spread of malaria among displaced communities in Africa. Nevertheless, these crucial endeavors encounter several impediments. The transient character of these settlements hinders the maintenance of health records and the monitoring of malaria cases, consequently posing difficulties in accurately evaluating the extent of the disease [56]. Moreover, the presence of disruptions in health information systems, resource limitations, and a lack of technical knowledge result in gaps and inaccuracies in malaria data from these specific settings [57, 58]. Security concerns and restricted access in specific areas can further impede the efforts to carry out comprehensive monitoring and collect crucial data [59]. The lack of standardized procedures and instruments for collecting data in displaced environments hinders the capacity to compare and merge malaria data from various areas [60, 61]. The inadequate coordination and data exchange across humanitarian organizations, government agencies, and health authorities exacerbates this scenario, leading to fragmented and unreliable malaria monitoring data [62].

The presence of displaced populations in places with insufficient healthcare infrastructure exacerbates the complications, making it difficult to employ advanced diagnostic and surveillance systems. Conventional diagnostic techniques, including examining blood smears under a microscope, require a lot of effort and specific expertise, and may not be easily accessible in rural or resource-limited areas [33]. As a result, there is a significant deficiency in the early detection and immediate treatment of malaria, which worsens the burden of the disease [51]. Additionally, the transient nature of these populations poses obstacles to conducting long-term studies and sustaining continuous monitoring. Tracking individual travels and ensuring consistent health records are particularly onerous tasks. To tackle these problems, it is necessary to recognize and overcome the practical, social, and financial obstacles that hinder the implementation and acceptance of technology in these areas.

### Current efforts and interventions

A coalition of international organizations, non-governmental organizations (NGOs), and local communities have launched coordinated efforts and interventions to address the significant problem of malaria among displaced populations in Africa. The continual challenges of ongoing conflict, significant population displacements, and limited healthcare accessibility present significant barriers to the effective execution of these programs [21]. These activities aim to improve the access and use of effective malaria prevention, diagnostic, and treatment services for refugees, IDPs, and other vulnerable groups living in temporary settlements or displacement camps [62, 63].

However, the treatments often face obstacles that hinder their impact and efficacy. These obstacles include limited resources, logistical difficulties, and the inherent problems of reaching and involving people who regularly move. This demonstrates a flexible, durable, and cooperative strategy for overcoming obstacles and substantially decreasing the prevalence of malaria in Africa's displaced populations.

#### Role of international and local organizations and malaria control programs

The malaria control landscape for displaced populations in Africa is shaped by the combined efforts of various international and local organizations alongside malaria control programs tailored to the unique needs of these vulnerable communities. The WHO and the UNHCR have collaborated extensively to deploy essential interventions such as long-lasting insecticidal nets (LLINs) and intermittent preventive treatment in pregnancy (IPTp) within refugee camps, albeit facing challenges like resource constraints and logistical complexities [22, 64, 65].

Another intervention is the WHO's "Roll Back Malaria" initiative which provides overarching technical guidance and support to national malaria control programs, bolstering global efforts to combat the disease [66]. Additionally, the UN Refugee Agency (UNHCR) and the International Organization for Migration (IOM) collaborate closely with host governments to ensure equitable access to malaria prevention, diagnosis, and treatment services among displaced populations [67].

At the local level, non-governmental organizations (NGOs) play a pivotal role in bridging service gaps by directly delivering interventions, conducting educational campaigns, and fostering community participation in vector control activities [68]. These collaborative endeavors reinforce healthcare systems in displacement settings, striving to alleviate malaria-related morbidity and mortality among vulnerable populations. Key interventions include the distribution of LLINs to ensure coverage and utilization among refugees and IDPs, IRS campaigns for additional vector control, and the implementation of IPTp and SMC programs tailored to safeguard pregnant women and children, respectively [69–71]. Moreover, efforts to enhance case management through the provision of RDTs and artemisinin-based combination therapies alongside community-based approaches like training community health workers (CHW) and establishing mobile clinics further strengthen malaria service delivery in hard-to-reach displacement contexts [72–74].

In the broader context of malaria control across Africa, the adoption and expansion of WHO-recommended case management and vector control tools, notably in countries like South Sudan, underscoring the commitment to achieving Millennium Development Goals (MDGs) while emphasizing the importance of measuring the impact of malaria control on reducing morbidity and mortality [20, 75]. Comprehensive assessments utilizing WHO standard procedures for Malaria Programme Review (MPR) have been instrumental in guiding evaluation processes, involving stakeholders and partners in consensus-building, thematic desk reviews, field evaluations, and subsequent implementation planning [20, 76].

## Diagnosis and surveillance of malaria among displaced people in africa

### Diagnosis and surveillance of malaria in africa

Diagnostically, the severity and high prevalence of malaria may require detailed clinical examination and laboratory investigation [77]. Culturally, laboratory diagnosis of malaria is performed with microscopic examination of thick and thin blood films stained with Geimsa stain [4]. Microscopy has been recognized by the WHO as the prime standard for malaria diagnosis [78], but the outcome is subjective, hence, there is a need for competent medical laboratory experts with extensive training and in-depth knowledge of detecting the malaria parasite microscopically. Regrettably, most non-endemic areas with the upsurge of imported malaria are characterized by limited laboratory technologists who are trained and experienced in identifying Plasmodium species [2].

This is significant because if performed by inexperienced individuals, particularly in areas where the disease is common, there is a risk of developing cases with low sensitivity [4] This can lead to either over- or under-diagnosis, resulting in the excessive use of anti-malaria drugs or neglecting treatment. Both of these scenarios can contribute to the facilitation and escalation of resistance by malaria-causing organisms or the occurrence of drug-induced pathological conditions. In addition, there have been reports of changes in the physical structure of parasites caused by excessive or incorrect use of drugs. These changes can lead to incorrect diagnoses of malaria, highlighting the importance of skilled microscopists who can accurately identify the species of parasites. This identification is crucial for effective treatment [2, 79]. Hence, it is imperative to assess the existing malaria diagnostic method in order to enhance the detection and monitoring of malaria in Africa, particularly among displaced populations. The classical method's limitations have led to the development of RDTs, which offer more convenience and speed compared to classical microscopy. However, research has indicated that the dependability of the results from RDTs can be inconsistent [80, 81]. RDTs provide more accurate diagnostic outcomes by identifying several antigens such as Plasmodium lactate dehydrogenase (pLDH), aldolase, and histidine-rich protein 2 (HRP2) throughout the erythrocytic phase of the parasite's life cycle [77].

At present, there are multiple methods for diagnosing malaria known as RDTs. However, the effectiveness of these methods can vary depending on factors such as the area, environment, and the presence of the disease and parasites [4]. Through the progress made in diagnostic science, researchers have identified several drawbacks of RDTs, including subpar product performance, inadequate quality control, and limited ability to address these deficiencies [82]. In order to improve the recognized constraints, scientists have conducted ongoing research that has resulted in significant advancements and discoveries in molecular techniques, particularly polymerase chain reaction (PCR) assays such as classical PCR, isothermal PCR, and real-time PCR [83]. Real-time PCR (RT-PCR) is advantageous due to its timeliness, reduced need for human resources, and ability to provide quantitative data [79, 84].

The utilization of molecular technologies in malaria diagnosis initiated the age of detecting malaria at a minimal level and identifying the specific species of the malaria parasite [85]. Nevertheless, the Plasmodium RT-PCR protocols, although seemingly advantageous, have certain drawbacks. These include limitations in species differentiation [79, 86], a restricted number of species identification [81, 87], and the need for prior genus identification and additional post-PCR analysis [88]. Consequently, these factors contribute to the prolonged duration of malaria diagnosis and surveillance.

Moreover, relying just on symptomatic persons who seek medical care for malaria treatment is not sufficient for successful malaria management, as asymptomatic individuals will not be identified [89]. Efficient methods such as mass screening and treatment (MSAT) and mass drug administration (MDA) have proven to be efficient in reaching those who do not show symptoms [90]. The MSAT methodology utilizes a resource-intensive method to identify active cases of malaria, employing a selective screening strategy similar to previous programs aimed at controlling infectious diseases [91].

The length, severity, and proximity to conflict or war zones are connected with higher rates of malaria parasites and a greater prevalence of malaria sickness [92]. This statement is highly accurate because conflict has a direct correlation with several risk factors, such as the failure of control programs, which can lead to an increase in malaria transmission [93]. Unfortunately, new geostatistical data shows that there has been a rise in these types of crises in sub-Saharan Africa, accompanied by other complexities [94]. An optimal strategy for controlling and eliminating malaria should prioritize the prompt identification and management of infections caused by the malaria parasite [17].

Given the substantial influence of war on the effectiveness of malaria control efforts, it is crucial to investigate ways that enhance the diagnosis and monitoring of malaria as a long-term intervention. The absence of surveillance for malaria identification, prevention, and control in Africa is closely associated with inadequate access to competent healthcare and the substandard diagnosis of malaria. The presence of data on the present diagnosis and surveillance would enhance comprehension of the relationship between malaria epidemiology and political, social, and economic instabilities, as well as local and national population displacements, in different African countries. Therefore, the insufficient information on the diagnosis and monitoring of malaria is the cause of the limited comprehension of the epidemiological data on malaria in Africa [95].

### Challenges facing effective surveillance of malaria among displaced people in Africa

Effective malaria surveillance remains a challenge, especially among the displaced people irrespective of the different diagnostic and surveillance methodologies, for example, PCRs and RDTs available on the African market. Most of these methodologies rely on systematic data and information collection relevant to epidemic prevention, which is often problematic and difficult to obtain in displacement settings [96]. These displacement settings are characterized by disruptions in routine health services, minimal resources for data collection, and aggregation, and poor coordination among different stakeholders, such as CHW. Besides, some of these displacement settings have cross-border scenarios that may have multiple governments and international agencies involved [97]. For example, Eshag et al. demonstrate a limited presence of resources and control options necessary for malaria elimination in some of the displacement sites in Darfur Sudan [9]. The absence of these resources was attributed to the unstable atmosphere characterized by conflicts and upheavals and the outcome was an increased malaria prevalence noted at a 61.2% prevalence proportion [9]. The political instability in these displacement sites creates significant barriers to the establishment of specific data collection methodologies, especially those that require attention to detail, such as the evaluation of the mosquito vector species [9].

**Fig. 2:**
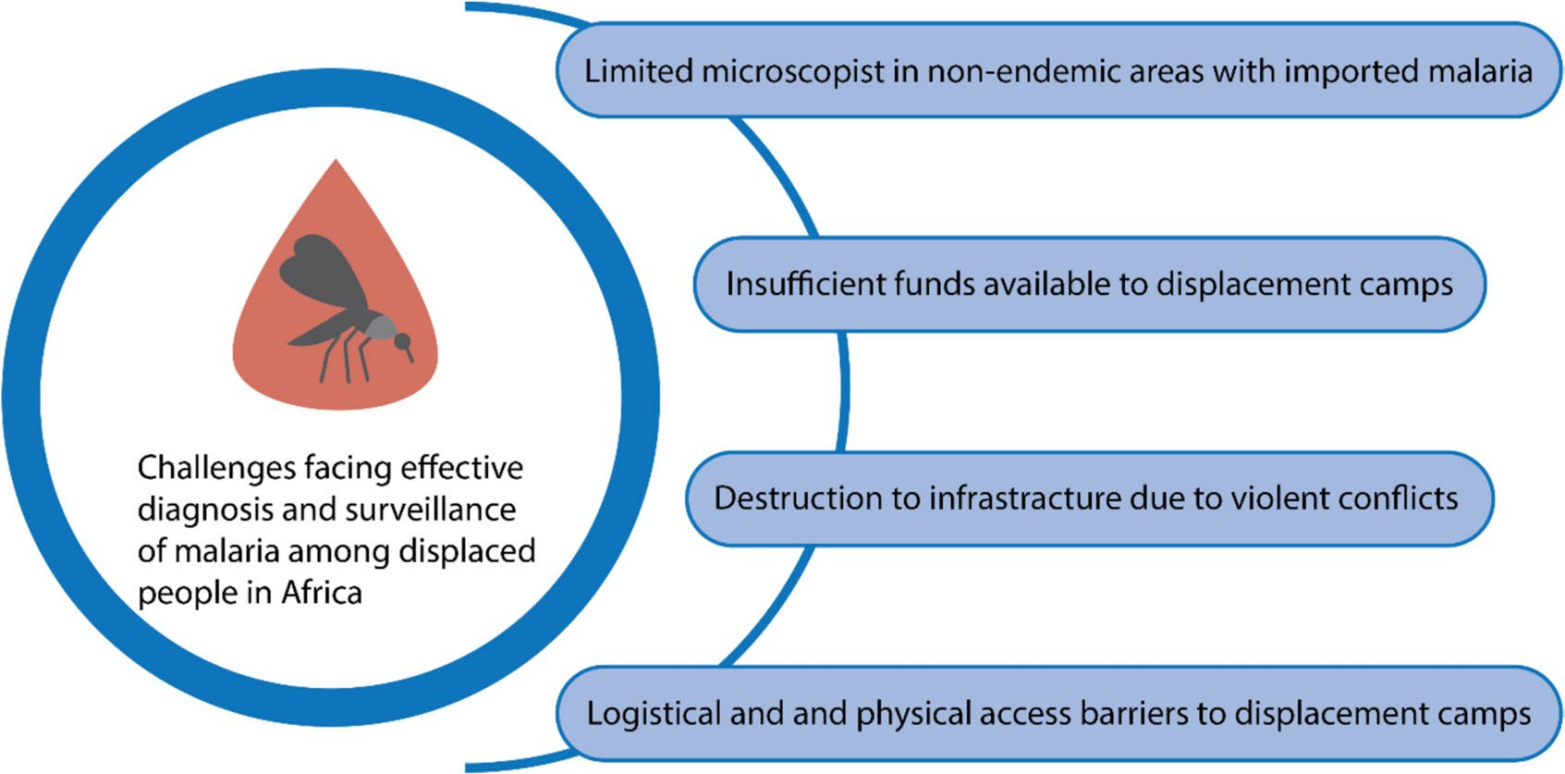
Challenges facing effective surveillance of malaria among displaced people in Africa

Moreover, malaria diagnosis and surveillance are particularly challenging in tropical regions facing CHEs, especially in war-torn zones housing IDPs. The negative impacts of violent conflicts, such as the destruction of infrastructure (Fig. 2.), impediments to transportation and delivery of medical supplies, the mass emigration of qualified medical personnel, and the overall displacement of populations, are well documented in the literature. These factors collectively hinder effective malaria surveillance and control in such settings [19]. These challenges cumulatively pose great threats to the healthcare system, worsen living conditions, deter access to basic healthcare, and heighten the risks and susceptibility to infectious diseases [19, 98]. As a result of the poor healthcare system, increased morbidity and mortality are experienced which are principally caused by infectious diseases like malaria in sub-Saharan Africa [27]. For instance, the DRC was reported to face an upsurge in malaria-induced morbidity and mortality during and after serious social conflicts [17]. Also, the Central African Republic has consistently been bedeviled with frequent violent conflicts and political crises over the years resulting in the displacement of residents. The worst hit is the impact on the healthcare system where medical essentials and drugs were looted from hospitals and other healthcare facilities throughout the country making more people to become vulnerable to infectious diseases [19]. A survey study documented a malaria-induced mortality increase of 3.5-fold from a total of 5.5-fold death increase during violent conflict [23].

Malaria surveillance is also hindered by logistical and physical access barriers among the displaced sites and populations. Often, individuals in communities that face upheavals and armed conflicts may relocate to remote and hard-to-reach areas with limited infrastructure, such as road networks. The DRC, for example, has approximately 2.7 million internally displaced people living in various displacement and settlement camps [18]. Some of these populations live in villages and camps, for example, Bilobilo camp and Mubi Village which are situated along the sparse equatorial Congo Forest and Lowa River [18]. The geographical location coupled with an unstable atmosphere characterized by rebel activity creates a major barrier concerning malaria surveillance. Notably, accessibility is not merely a geographical issue but also a political and security-related one, particularly in conflict zones where the risk of violence can prevent public health officials from carrying out malaria surveillance and control activities.

Funding availability to the different camps housing displaced people has been critical in determining the magnitude of malaria surveillance. Often, the surveillance and vector control methodologies are constrained in the absence of sufficient finances that are relevant during the procurement process and offset health worker wages. For example, between 2020 and 2021, a community surveillance project to determine the prevalence and test-positivity of malaria among 12 resettlement sites was implemented by the Mozambican Ministry of Health [12]. The surveillance strategy was particularly funded by the Norwegian government and further supported by volunteer doctors from the "University College for Aspiring Missionary Doctors Africa." The project demonstrated a test-positivity of 23.3% and 58.2% among individuals living in Sofala and Cabo Delgado provinces in Mozambique [12]. Further, the project categorically described the spatial–temporal patterns of malaria distribution in these provinces offering substantial information to the Mozambican Ministry of Health.

The presence of external support in the form of this funding demonstrates the impact that can be achieved concerning malaria surveillance in African nations. With these prevalence and spatial–temporal trends, different national strategies can be effectively developed to overcome the epidemic and improve the quality of life of individuals. However, most of the African countries are constrained by their budget allocations, and some lack access to external funding creating barriers to effective malaria surveillance. Additionally, humanitarian health interventions, especially in resettlement camps, often operate under emergency conditions with short-term and unpredictable funding [74]. Thus, long-term investments, which are crucial for sustainable malaria control, are frequently insufficient and unavailable. Additionally, financial uncertainty affects the procurement of necessary supplies like ITNs, antimalarial drugs, and diagnostic equipment impeding successful surveillance. These challenges create significant public and global health concerns hindering the achievement of global targets, such as the "global technical strategy" which focuses on a mortality reduction of 90% by 2030, the "global vector control response 2017–2030 strategy," and the "Sahel Malaria Elimination Initiative" as set by the WHO [99].

### Effect

Inadequate surveillance of malaria among displaced populations exacerbates the risk of malaria infection. The absence of effective surveillance systems makes it difficult to detect and treat cases early, leading to higher rates of severe malaria and fatalities. For example, a study conducted in a refugee settlement in Sub-Saharan Africa found that children from displaced communities who were hospitalized with severe malaria exhibited more severe symptoms and were over twice as likely to die compared to local children [16]. This was often attributed to delayed access to medical care, geographical obstacles, and logistical challenges, with distance to healthcare facilities being a factor in a quarter of cases.

**Fig. 3:**
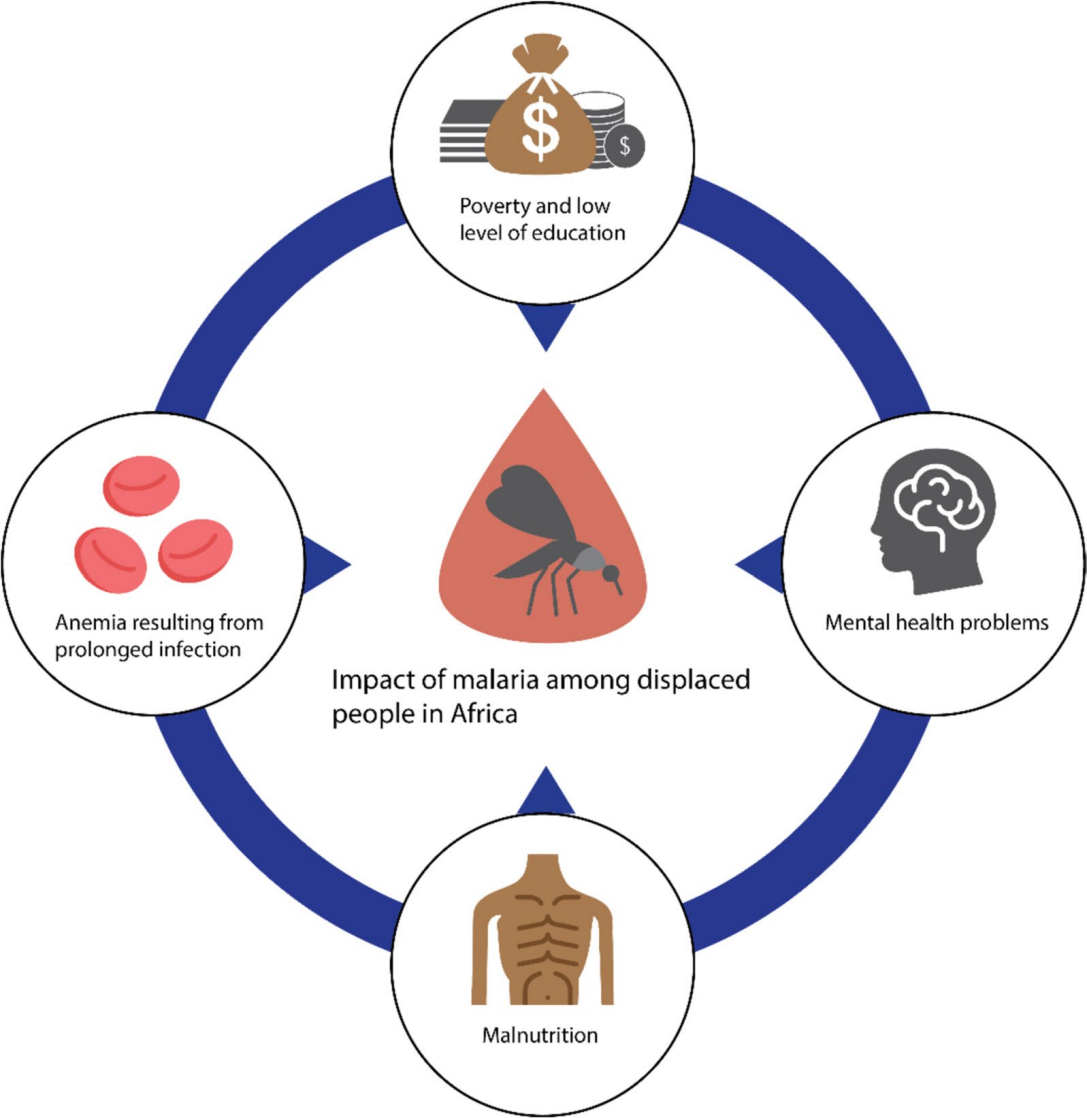
Impact of malaria on displaced people in Africa

The primary causes of malaria-related fatalities included anemia resulting from prolonged infection, malnutrition (Fig. 3), and parasitic worm infections [16]. Furthermore, insufficient monitoring of malaria treatment in refugee settings may contribute to the development of drug-resistant malaria strains, adding complexity to treatment efforts and burdening already strained healthcare systems [100]. The economic ramifications of high malaria prevalence among displaced populations are profound. These groups already occupy lower rungs on the economic ladder, and studies have indicated that their low socioeconomic status can perpetuate malaria transmission and its associated burden [101].

Malaria treatment is financially draining, especially among displaced families, who are already struggling with limited resources. Some of the minimal resources are mostly redirected to, sourcing basic needs, such as food and shelter. Thus, the absence of enough resources allocated for managing the disease condition limits the promotion of adequate healthcare. The health effects of malaria among displaced communities are very severe, resulting in higher rates of illness and death. Witnessing loved ones suffer or succumb to malaria can exacerbate trauma and mental health issues, particularly among vulnerable groups such as children and survivors of conflict or natural disasters [102].

As expected, displacement also frequently leads to restricted healthcare access and insufficient preventive measures, intensifying the disease's impact, however, improved diagnostic methods and efficient monitoring systems are crucial for managing the disease, as they allow for early detection and treatment. This is essential for minimizing the number of complications and deaths caused by malaria in these areas [102]. Displaced people in Africa are also affected socio-economically through certain events, policies, or actions on the social and economic aspects of a society that displacement brings with it coupled with the fight against malaria [101].

Illness-induced decrease in output, along with the financial costs of treatment, can sustain poverty in people who have been forced to leave their homes, where there are already few economic options available, the additional burden of malaria can have a severe impact on the financial stability of both individuals and families but enhancing diagnostic methods and disease monitoring can lead to improved health results, potentially mitigating the economic consequences of malaria on these communities [101].

Also, the psychological effects of malaria extend beyond the immediate signs of the disease. The constant possibility of transmission creates an atmosphere of anxiety and uncertainty, especially among vulnerable populations like displaced youngsters and pregnant women. The mental well-being of individuals in displaced communities can be significantly impacted by anxiety, stress, and trauma resulting from this dread, leading to wide-ranging implications that can increase the burden of malaria infection among displaced populations. The uncertainty surrounding access to timely healthcare produces a pervasive sense of anxiety and helplessness [102]. A study undertaken by the WHO revealed that pregnant women who contract malaria exhibit more severe symptoms and have greater risks of negative outcomes, including miscarriage, intrauterine demise, premature delivery, low-birth-weight neonates, and neonatal death. This underscores the pressing need for enhanced diagnostic and monitoring techniques to detect and manage malaria among displaced African communities [19], especially among displaced pregnant women who have a high risk of infection [103].

Enhancing diagnostic and surveillance technologies would not only facilitate the detection and treatment of malaria cases among displaced populations but also assist in tracking the spread of the illness [16]. Over time, enhanced surveillance techniques have resulted in the timely identification of malaria epidemics, facilitating immediate intervention and preventive actions in developed countries. Thus incorporating these tactics into Africa’s current healthcare systems, especially amongst displaced people will improve the overall administration and regulation of malaria [102].

To effectively combat malaria among displaced people in Africa, it is essential to implement comprehensive measures that incorporate improved diagnosis methods, surveillance systems, and socio-psychological assistance. Immediate measures are required to alleviate the condition's effects, considering its connection with socioeconomic inequalities, physiological, and health challenges. Also, effective collaboration between the healthcare, policy, and humanitarian sectors is crucial for developing targeted interventions that prioritize the health and well-being of vulnerable people. Finally, allocating resources to comprehensive strategies not only preserves lives but also promotes resilient and fair societies where access to healthcare is considered a fundamental human right [102].

## Recommendations

Despite the implementation of control programs, malaria remains a significant health concern among displaced populations in Africa. Improving diagnostics and surveillance in these settings necessitates a comprehensive approach that addresses the unique challenges these communities face. The UNHCR strategic plan highlights the importance of integrating refugee-specific objectives into national malaria control plans, recognizing the unique barriers such as limited healthcare access, environmental conditions, and socioeconomic factors that influence malaria transmission [104].

Additionally, the lack of governmental institutions in many settings of displacement dictates the necessity of creating alternative local institutions that are sustainable. These have to aim at long-term support for the displaced populations, especially in addressing their health challenges. Building resilient and adaptable local frameworks allows for filling the gap left by governmental organizations in ensuring continued assistance for vulnerable groups.

Nevertheless, investment in the training and rehabilitation of social workers will be needed to meet the needs of long-term diagnostic and follow-up methods. Encouragement of academic studies and professional development among both local host communities and displaced populations will enhance the capacity for ongoing health education and awareness. Social workers can ensure the survival and continuity of awareness and educational programs on societal issues among the displaced, leading to a sustainable health support system.

Active community engagement is crucial for raising awareness about malaria and its prevention. This can be achieved through community meetings, educational sessions, and the distribution of informational materials in local languages. By involving the community in awareness campaigns, they become more proactive in mitigating the spread of malaria. Utilizing CHWs plays a vital role in enhancing malaria surveillance and diagnostics in displaced settings. CHWs can conduct door-to-door screenings, distribute mosquito nets, and provide primary treatment for uncomplicated malaria cases. Their familiarity with community dynamics enables them to reach remote areas where formal healthcare services may not be accessible [18].

Routine surveillance data is essential for estimating malaria burdens and guiding control efforts. Leveraging such data helps define national strategic targets, assess progress in malaria control, and identify areas requiring increased intervention and resources. Furthermore, such data can help in training artificial intelligence, such as utilizing mathematical models to predict the patterns of transmission and severity of the disease among IDPs. RDTs have significantly improved diagnostic accuracy, particularly in high-risk areas [105, 106]. However, challenges such as variability in RDT sensitivity and parasite mutations affecting test efficacy need to be addressed to enhance diagnostic reliability.

Environmental management is also crucial when resettling displaced populations in previously uninhabited, malaria-prone areas. Resettlement may increase proximity to mosquito breeding sites, particularly in deforested areas. Effective environmental management should include reforestation, proper drainage systems to eliminate stagnant water, and community-led initiatives to reduce mosquito breeding sites.

Integrating malaria control efforts with other health services, such as reproductive health, maternal and child health, and nutrition programs, can improve access to comprehensive healthcare. This approach promotes efficiency, reduces duplication of efforts, and maximizes the impact of limited resources. Given the unpredictable nature of displacement settings, healthcare interventions must be flexible and adaptive. Strategies should be agile enough to respond to changing circumstances, such as conflict-induced population movements, natural disasters, or infectious disease outbreaks like COVID-19, without compromising the continuity of malaria services.

Mobile health technologies, including apps for symptom reporting, remote consultations, and RDT kits, are crucial for mitigating malaria risk among refugees relocating from low-transmission to high-transmission areas. These tools facilitate real-time monitoring of malaria cases, enabling fast response interventions and resource allocation based on emerging trends and hotspot identification [17]. Furthermore, policies for malaria diagnosis should incorporate both microscopy and RDT kits for higher accuracy. Integrating microscopy (high specificity) with RDT (high sensitivity) minimizes false positives and negatives. Adequate training and quality assurance mechanisms should be in place to ensure consistent and reliable diagnostic outcomes [4].

Decentralizing primary healthcare to the community level, facilitated by a network of CHWs, can effectively manage malaria in hard-to-reach, conflict-affected populations experiencing frequent displacements. This approach can significantly improve health outcomes in these areas [19]. By addressing these key areas, it is possible to improve malaria diagnostics and surveillance in displaced populations, ultimately reducing the disease burden and enhancing health outcomes in these vulnerable communities Table 4.highlights the aforementioned key strategies for improving malaria diagnostics and surveillance among displaced populations in Africa.

**Table 4:** Key strategies for improving malaria diagnostics and surveillance among displaced populations in Africa

Key Area	Details
*Community Engagement*	Raising awareness through community meetings, educational sessions, and distribution of informational materials in local languages
*Utilization of Community Health Workers (CHWs)*	CHWs conduct door-to-door screenings, distribute mosquito nets, and provide primary treatment for uncomplicated malaria cases. They can reach remote areas where formal healthcare services may not be accessible
*Routine Surveillance Data*	Essential for estimating malaria burdens and guiding control efforts. Helps define national strategic targets, assess progress, and identify areas needing increased intervention and resources
*Environmental Management*	Crucial in resettling populations in malaria-prone areas. Includes reforestation, proper drainage systems, and community-led initiatives to reduce mosquito breeding sites
*Integration of Health Services*	Combining malaria control efforts with reproductive health, maternal and child health, and nutrition programs. Promotes efficiency, reduces duplication of efforts, and maximizes the impact of limited resources
*Flexible and Adaptive Healthcare Interventions*	Strategies must respond to changing circumstances, such as conflict-induced movements, natural disasters, or disease outbreaks like COVID-19, without compromising malaria services
*Mobile Health Technologies*	Use of apps for symptom reporting, remote consultations, and RDT kits. Facilitates real-time monitoring, fast response interventions, and resource allocation based on emerging trends and hotspot identification
*Combination of Diagnostic Methods*	Incorporating both microscopy and RDT kits for higher diagnostic accuracy. Adequate training and quality assurance mechanisms should ensure consistent and reliable outcomes
*Decentralized Primary Healthcare*	CHWs manage malaria in hard-to-reach, conflict-affected populations experiencing frequent displacements

## Limitations

Despite thorough searches across multiple databases, the accessibility of literature may remain limited, with the possibility of overlooking relevant studies not indexed in the selected databases. Furthermore, language bias may exist due to restricting the inclusion criteria to articles published in English, potentially excluding pertinent studies published in other languages and leading to an incomplete representation of the literature. The potential for publication bias should also be considered, as studies reporting positive or statistically significant results are more likely to be published, potentially skewing the overall findings.

## Conclusion

Despite recent progress toward malaria elimination in various African countries, malaria remains a significant health problem among displaced populations, particularly in regions with weak healthcare systems. Diagnostic strategies for malaria have seen little innovation over the past three decades, with microscopy still being the primary method used in many diagnostic laboratories. There is a pressing need for more rapid tests that do not compromise sensitivity and can be utilized in both medical facilities and resource-limited field settings. Displaced populations are particularly vulnerable to malaria, with a substantial risk of epidemics emerging in IDP sites. To address this, the WHO should develop and disseminate standardized operating procedures for malaria detection and surveillance tailored to the needs of displaced people in Africa. These strategies must be adaptable to specific locations and should prioritize access to essential health services and malaria-related commodities. By implementing accessible and efficient strategies, global malaria detection can become more sensitive, enabling faster and more targeted responses for disease control.

## Data Availability

No datasets were generated or analysed during the current study.

## Abbreviations

IDPs: Internally Displaced Persons
CHW: Community Health Workers
WHO: World Health Organization
UNHCR: United Nations High Commissioner for Refugees
DRC: Democratic Republic of Congo
ITN: Insecticide-Treated Nets
IRS: Indoor Residual Spraying
IPT: Intermittent Preventive Treatment
IPTp: Intermittent Preventive Treatment in Pregnancy
SMC: Seasonal Malaria Chemoprevention
LLIN: Long-Lasting Insecticidal Nets
RDT: Rapid Diagnostic Tests
PCR: Polymerase Chain Reaction
MSAT: Mass Screening and Treatment
CHE: Complex Humanitarian Emergencies
